# Explainable machine-learning integration of pre-task and preparatory EEG: revealing complementary predictive information for expert action reliability

**DOI:** 10.3389/fnins.2026.1907378

**Published:** 2026-07-30

**Authors:** Qiwei Zhao, Chenglin Zhou

**Affiliations:** School of Psychology, Shanghai University of Sport, Shanghai, China

**Keywords:** alpha oscillations, brain-behavior integration, EEG, explainable machine learning, motor expertise, preparatory activity, table tennis

## Abstract

**Introduction:**

Expert motor behavior varies among athletes and across repetitive actions; however, conventional brain-behavior analyses can conflate these statistical levels. We characterized the behavioral architecture of expert table tennis serves and tested whether pre-task and action-preparatory EEG contribute information at different behavioral scales.

**Methods:**

A total of 22 expert athletes completed 100 cue-directed serves with trial-resolved landing coordinates. Unsupervised principal component analysis characterized complete landing distributions. Eyes-closed pre-task EEG provided a hypothesis-informed alpha-organization index combining individual alpha peak frequency and fronto-central alpha coherence. EEG spectral power during action preparation was evaluated using nested leave-one-athlete-out learning, within-athlete permutation testing, grouped permutation importance, and multiscale probability integration.

**Results:**

The two dominant landing components explained 57.2% of behavioral variance and primarily reflected reproducible individualized placement patterns, whereas a smaller component (7.4% explained variance) was more closely aligned with task-defined performance. Pre-task alpha organization was associated with higher target-zone reliability (*r* = 0.68, *p* = 0.002) and a smaller 95% landing area (*r* = −0.50, *p* = 0.034; *n* = 18). Preparatory EEG contained modest information that generalized trial outcomes to unseen athletes (macro AUC = 0.589; *p* = 0.0063), with predictive information concentrated in theta activity in both preparation stages, Prep2 high-beta activity, and parietal sensors. Athlete-average preparatory activities did not reconstruct pre-task alpha organization under leave-one-athlete-out validation.

**Discussion:**

Pre-task and preparatory EEG carried complementary rather than interchangeable predictive information. Pre-task EEG was related to between-athlete performance propensity, whereas preparatory EEG offered weak but reliable within-athlete trial evidence. The value of the transparent machine-learning framework did not lie solely in achieving high prediction accuracy; rather, it was its capability to discover behavioral structure, test cross-athlete generalization, explain predictive information, and integrate neural evidence at a statistical level relevant to its operation.

## 1 Introduction

Expert motor performance is reliable without being mechanically identical. Skilled performers preserve task goals while exploiting multiple coordination solutions, a principle captured by motor abundance and repetition without repetition (Krakauer and Mazzoni, 2011; Ranganathan et al., 2020; Wolpert et al., 2011). This creates a measurement problem: large behavioral differences can reflect personalized style rather than superior control, whereas smaller variations can determine whether an action satisfies the task.

This variability is also hierarchical. Some differences are expressed between athletes as relatively persistent performance propensities or styles, whereas others arise within the same athlete from trial to trial. These levels answer different scientific questions: who is generally more reliable, and which action is more likely to succeed for a given athlete? Neural predictors must therefore be evaluated at the statistical level of the behavior they are intended to explain. Pooling athletes and trials can conflate stable individual differences with transient action-specific variation.

Table tennis serving exposes this hierarchy clearly. Athletes must direct the ball toward a cue-defined half-table target, but successful serves can still differ in depth, lateral bias, and dispersion. Landing coordinates can therefore quantify reliability (i.e., whether the instructed target zone is reached), spatial compactness, and finer directional calibration. Previous EEG studies of table tennis and self-paced sport have linked expertise and performance to alpha activity, functional coupling, and altered cortical recruitment during anticipation, preparation, or execution (Del Percio et al., 2009, 2011; Gallicchio et al., 2016; Raman and Filho, 2024; Studnicki and Ferris, 2023; Visser et al., 2022; Wolf et al., 2014, 2015). However, studies did not establish how neural organization measured before the task and neural activity preceding an individual’s action relate to different levels of behavioral variability.

Pre-task resting EEG and event-related preparation address distinct questions. Eyes-closed EEG provides a relatively low-artifact estimate of the baseline oscillatory organization. Individual alpha peak frequency (IAPF) characterizes a local oscillatory property and has been linked to attentional and cognitive processing; in contrast, alpha coherence quantifies large-scale coupling between areas (Grandy et al., 2013a; Grandy et al., 2013b; Haegens et al., 2014; Klimesch, 1999, 2012). Together, these measures provide complementary descriptions of oscillatory organization at local and network scales. In athletes, eyes-closed baseline IAPF has been positively associated with multiple-object tracking accuracy and was higher in elite than intermediate ice-hockey players (Zhang et al., 2021). However, baseline IAPF did not differentiate shooting-performance groups in a more homogeneous athlete sample (Christie et al., 2017). Therefore, pre-task EEG is regarded in this context as a candidate session baseline, rather than an immutable trait or a validated performance biomarker.

In contrast to pre-task EEG, neural activity during action preparation can vary substantially across trials and may contain information about the upcoming action. Previous studies have linked preparatory theta, alpha, and beta dynamics to motor planning, attentional allocation, and target-directed behavior (Bae and Luck, 2018; Cooke, 2013; Min and Park, 2010). In externally cued ice-hockey shooting, theta, alpha, and lower-beta dynamics also differed before and after target presentation, underscoring the need to preserve cue-relative preparation stages (Christie et al., 2019).

This hierarchical structure also constrains how predictive analyses should be conducted. The challenge is not maximizing classification accuracy, but evaluating neural information at the behavioral level where it operates. Machine learning is useful here not because a complex classifier is intrinsically preferable, but because it allows neural information to be evaluated at the behavioral level where it is expected to operate (Varoquaux, 2018; Woo et al., 2017; Wu et al., 2023; Yarkoni and Westfall, 2017). Multivariate time-resolved analysis can recover distributed neural information that is not apparent from any single sensor or feature (Grootswagers et al., 2017).

We hypothesized that expert serving behavior is organized across multiple statistical scales, and that neural predictors should be evaluated at the behavioral scale they are intended to explain. This was treated as an organizing assumption: pre-task EEG was evaluated in relation to athlete-level performance, whereas preparatory EEG was evaluated in relation to trial-level variation among individuals. We first characterized the dominant structure of behavioral variability, tested these two EEG-behavior links, and finally evaluated whether the two neural measures reflected a common cross-timescale axis or complementary predictive information.

## 2 Materials and methods

### 2.1 Participants

The analyzed expert cohort comprised 22 table tennis athletes, each contributing 100 serve trials (2,200 trials in the complete behavioral dataset). Four athletes were excluded from EEG analyses because the corresponding EEG data were unavailable or unusable; thus, 18 athletes were finally included (14 men and 4 women, all right-handed; age: mean = 20.88 years, SD = 1.86, range 19–24; training years: mean = 11.67 years, SD = 2.67, range 6–16). All participants provided written informed consent before data collection. The study protocol was reviewed and approved by the local ethics committee (No. 102772023RT028) and was conducted in accordance with approved institutional procedures.

### 2.2 Experimental procedure

**Figure 1:**
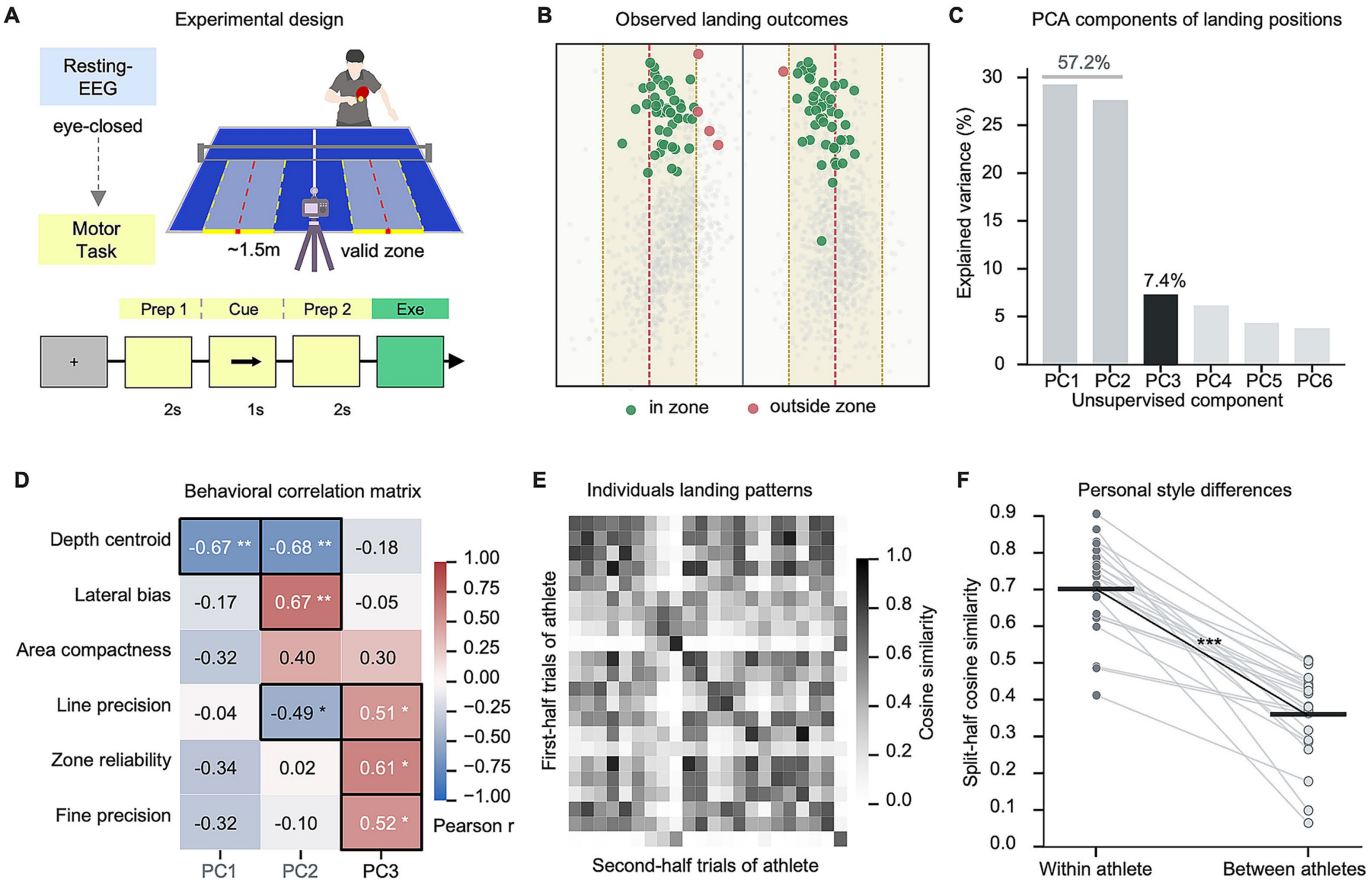
Experimental design and behavioral results. **(A)** Experimental design. **(B)** On-table landing coordinates on the calibrated 1,525 × 1,400 mm half-table. All athletes are shown in light gray; one example athlete is overlaid with target-zone landings in green and outside-zone landings in red. **(C)** Variance explained by unsupervised PCA of complete landing distributions. **(D)** Descriptive correlations used to interpret PC1-PC3; outlined cells and asterisks indicate within-component Benjamini-Hochberg-adjusted significance. *q < 0.05, **q < 0.01. **(E)** Split-half cosine similarity matrix. **(F)** Paired athlete-level observations comparing within-athlete and between-athlete similarity; ***p < 0.001 by permutation test.

As shown in Figure 1A, participants first completed 2 min of eye-closed pre-task EEG while seated and instructed to remain relaxed and minimize movement. The subsequent motor tasks comprised 100 cue-directed serves. Each trial began with a fixation and a 2-s preparation interval (Prep1) for directional information, followed by a 1-s left/right visual cue, a second 2-s informed preparation interval (Prep2), and action execution. Prep1 therefore indexed general preparation before the target direction was known, whereas Prep2 occurred after the athlete could incorporate the spatial goal. The cue interval itself was not treated as the principal motor-preparation period.

### 2.3 EEG acquisition

EEG was acquired with a portable QUICK-20R dry electrode system (Cognionics Inc., San Diego, CA, USA). The recordings contained 19 scalp channels positioned according to the extended international 10–20 system: F7, Fp1, Fp2, F8, F3, Fz, F4, C3, Cz, C4, T3, T4, P7, P3, Pz, P4, P8, O1, and O2. The original files also contained an A2-labeled auxiliary channel, one external channel, three-axis accelerometry, a packet counter, and a digital trigger stream; these auxiliary signals were not used as EEG predictors. Continuous signals were digitized at 1,000 Hz and stored in BrainVision Data Exchange format. The portable dry-electrode configuration was selected to permit recording in a behavioral testing environment. Such systems increase ecological feasibility but also require explicit artifact control and cautious sensor-level interpretation (Cheron et al., 2016; Tan et al., 2019; Wang et al., 2019).

### 2.4 Behavioral outcomes and calibrated table geometry

Serve landing positions were extracted from video recordings using Tracker, an open-source video-analysis tool built on the Open-Source Physics framework (Tracker, Open Source Physics). For each trial, an analyst inspected the recording frame by frame, identified the frame in which the ball contacted the table, and manually marked the center of the ball at the landing location. Pixel coordinates were subsequently calibrated to the physical dimensions of the recorded half-Table (1,525 × 1,400 mm) and expressed in millimeters (Figure 1B). Trials in which no valid table contact could be identified were coded as off-table or unsuccessful outcomes and were not assigned an on-table landing coordinate. Within each left or right half-table, the red line marked the instructed landing line and the middle half of that region, bounded by yellow lines, defined the target zone. Zero-coded, missing, or off-table outcomes were classified as failures and were excluded from spatial plots, but they remained in the denominator of target-zone reliability. *Reliability* was the number of target-zone landings divided by all trials. *Athlete-level compactness* was the area of the 95% bivariate ellipse fitted to target-zone landings; smaller values indicated tighter spatial control. *Line precision* was the performance-aligned inverse of absolute lateral distance from the instructed line, and *fine precision* summarized within-zone line accuracy. Exploratory trial-level outcomes additionally included absolute line error, inward-normalized line error, and leave-one-out radial compactness.

### 2.5 Unsupervised behavioral decomposition

For each athlete, cue-centered two-dimensional landing histograms and cue-specific failure mass were concatenated into a complete landing-distribution vector. Principal component analysis (PCA) was estimated without EEG variables or performance labels (Jolliffe and Cadima, 2016). The components were therefore treated as descriptive axes of landing-distribution variance rather than as predefined style or performance factors. They were interpreted after estimation by Pearson correlations with *depth centroid, lateral bias, area compactness, line precision, zone reliability,* and *fine precision*. Benjamini–Hochberg false-discovery rate adjustment within each component was used for annotations in Figure 1D. Split-half cosine similarity quantified whether the first and second halves of an athlete’s landing distribution were more similar within an athlete than between athletes. Bidirectional nearest-neighbor matching and label permutation tested whether the distributions contained reproducible identity information.

### 2.6 Pre-task EEG preprocessing and alpha organization

Only eye-closed pre-task recording was used for the primary baseline analysis because it minimized movement contamination and provided a favorable state for estimating alpha peaks and resting coupling. The 19 scalp channels were selected, average-referenced, notch-filtered at 50 Hz, and band-pass filtered from 1 to 45 Hz. Samples exceeding four channel-wise standard deviations from the channel median were marked; sparsely marked samples (<10% of a channel) were linearly interpolated, whereas channels with more extensive contamination were excluded from that recording. Power spectral density was estimated with Welch’s method using 4-s windows and 50% overlap. IAPF was obtained from the cleaned alpha-range spectrum. Magnitude-squared coherence was computed at 8–12 Hz and averaged over all available pairs linking frontal sensors (Fp1, Fp2, F7, F8, F3, Fz, F4) to central/temporal sensors (C3, Cz, C4, T3, T4). A hypothesis-informed composite alpha-organization index was defined as z(IAPF)–z(fronto-central alpha coherence), such that higher values indicated a faster alpha peak and lower broad fronto-central coupling.

### 2.7 Trial-level preparation-EEG preprocessing

EEG data during preparation and action execution were notch-filtered at 50 Hz, band-pass filtered from 1 to 45 Hz, average-referenced, and resampled to 250 Hz. Channels with robust z scores > 4 for log channel standard deviation, or near-zero variance, were marked as bad; the median was 1 channel per recording (range 0–4). Within each epoch, marked channels were replaced by the time-varying mean of usable channels. Prep1 and Prep2 were trimmed by 10% at each boundary to reduce transition contamination. Epochs were rejected when within-athlete-and-stage robust z scores of log peak-to-peak amplitude or log root-mean-square amplitude exceeded 4.5, or when more than 35% of channels were unavailable. Across the aligned stage epochs, 98.6% passed these criteria. The primary feature space also excluded Fp1, Fp2, T3, and T4 to reduce sensitivity to ocular and temporal muscle activity.

### 2.8 Spectral features and nested cross-athlete learning

Welch spectra were calculated for each retained Prep1 and Prep2 epoch using approximately 1-s segments with 50% overlap. Absolute power was integrated over theta (4–7 Hz), alpha (8–12 Hz), low beta (13–20 Hz), and high beta (20–30 Hz), then log10-transformed. Then the representation comprised 15 sensors × 4 bands × 2 stages = 120 features. The binary target was whether the serve entered the cue-specific target zone. The outer validation loop left one athlete out. Within the remaining athletes, grouped cross-validation selected 20 or 50 univariate features and a regularization parameter (C = 0.05 or 0.20) for an L2-regularized, class-balanced logistic regression. Median imputation, standardization, feature selection, and hyperparameter selection were all fitted exclusively to training athletes. Performance was computed as AUC within each held-out athlete and then averaged without trial-count weighting (macro AUC). A fixed 10,000-iteration within-athlete label-permutation test preserved athlete-specific hit rates and tested the complete out-of-sample prediction vector (Hastie et al., 2009; Nichols and Holmes, 2002).

### 2.9 Explainability and multiscale integration

Grouped permutation importance was evaluated only for each held-out athlete. All features belonging to a stage-by-band or stage-by-region group were jointly shuffled 100 times, and importance was the resulting decrease in held-out AUC. Athlete-bootstrap 95% confidence intervals summarized uncertainty across the 18 evaluable outer folds; one-sided sign-flip tests quantified whether fold-wise importance was consistently positive. These exploratory group tests were not multiplicity-corrected. Importance, therefore, identifies the information required by the fitted predictor, not a causal neural generator.

For multiscale integration, the pre-task alpha organization supplied an athlete-level baseline probability. Cross-fitted preparation predictions were centered on an athlete to represent trial evidence relative to that athlete’s own mean. A second-level logistic model combined the two predictors using training-fold information only. Pooled AUC assessed discrimination across all trials, macro AUC assessed within-athlete ranking, and the Brier score assessed probabilistic calibration. Fold-wise preparation coefficients tested whether trial evidence entered the combined model in a consistent direction. Finally, stage-by-band-by-region preparation spectra were averaged within each athlete. Nested leave-one-athlete-out ridge regression was used to test whether these averages could reconstruct pre-task alpha organization or overall reliability. Negative cross-validated R2 values indicated failure to outperform an intercept-only prediction for the held-out athlete; ridge regularization was used to stabilize these high-dimensional regressions (Hoerl and Kennard, 1970).

### 2.10 Statistical analysis and software

Athlete-level associations used two-sided Pearson correlations. Partial correlations controlled PC1 and PC2 scores, and leave-one-athlete-out recomputation assessed influence rather than external generalization. Unless otherwise stated, uncertainty intervals are percentile bootstrap intervals with the athlete as the independent resampling unit. Sensitivity analyses evaluated stage-specific features, full Prep1-cue-Prep2 trajectories, continuous landing errors, and probabilistic landing models without redefining the primary endpoint. Analyses were implemented in Python with MNE-Python, SciPy, pandas, statsmodels, and scikit-learn (Gramfort, 2013; Gramfort et al., 2014; Pedregosa et al., 2011).

## 3 Results

### 3.1 Dominant landing variance reflected individualized organization

The PCA of complete landing distribution showed that PC1 and PC2 explained 29.4 and 27.7% of variance, respectively (57.2% combined), whereas PC3 explained 7.4% of variance (Figure 1C). PC1 and PC2 were most strongly associated with the depth centroid and lateral bias. PC3 showed stronger descriptive associations with zone reliability (*r* = 0.61), line precision (*r* = 0.51), and fine precision (*r* = 0.52; Figure 1D). Thus, in this dataset, the dominant landing-distribution components were more closely aligned with placement tendencies than with explicit reliability and precision. Complete landing distributions are reproducible. Split-half similarity was greater within athletes (cosine similarity = 0.70) than between athletes (0.36), and bidirectional top-1 identification reached 40.9% compared with 4.5% chance (permutation *p* < 0.001; Figures 1E,F).

### 3.2 Pre-task alpha organization related to athlete-level reliability and compactness

**Figure 2:**
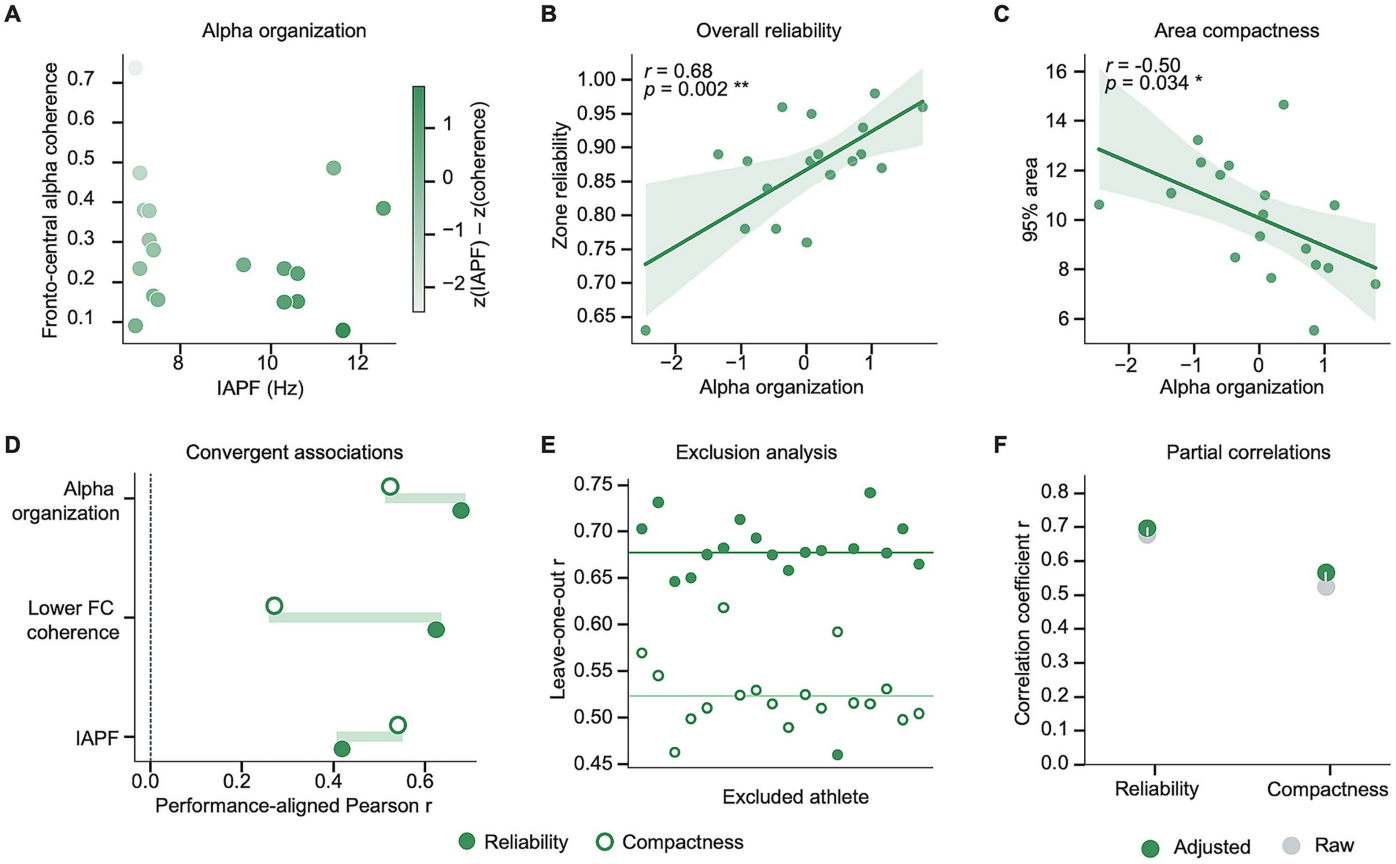
Pre-task alpha organization. **(A)** IAPF and fronto-central alpha-coherence space; Color indicates *z*(IAPF)–*z*(coherence). **(B,C)** Associations with target-zone reliability and 95% landing area. Shading denotes 95% confidence intervals. **(D)** Performance-aligned correlations for IAPF, lower fronto-central coherence, and their standardized contrast. **(E)** Correlations recomputed after excluding each athlete; Horizontal lines show the full-sample estimates. **(F)** Raw correlations and partial correlations adjusted for PC1-PC2 placement style. Leave-one-athlete-out analysis assesses influence, not external predictive performance.

Alpha organization was the standardized contrast between higher IAPF and lower fronto-central alpha coherence (Figure 2A). Across the 18 athletes with complete pre-task estimates, higher alpha organization was associated with greater target-zone reliability (*r* = 0.68, *p* = 0.002) and smaller 95% landing area (*r* = −0.50, *p* = 0.034; Figures 2B,C).

The two constituents showed directionally convergent but weaker associations (Figure 2D). For reliability, IAPF correlated at *r* = 0.42 and performance-aligned lower fronto-central coherence at *r* = 0.59, compared with *r* = 0.68 for their standardized contrast. Leave-one-athlete-out estimates remained positive for both outcomes across all exclusions, although compactness was less stable (Figure 2E). Adjustment for PC1 and PC2 placement-style scores did not attenuate the results (partial r = 0.70 for reliability and 0.57 for compactness; Figure 2F). The index therefore summarizes a candidate pre-task baseline in this cohort, although its broad validity remains to be established.

### 3.3 Preparatory EEG contained generalizable and interpretable trial information

**Figure 3:**
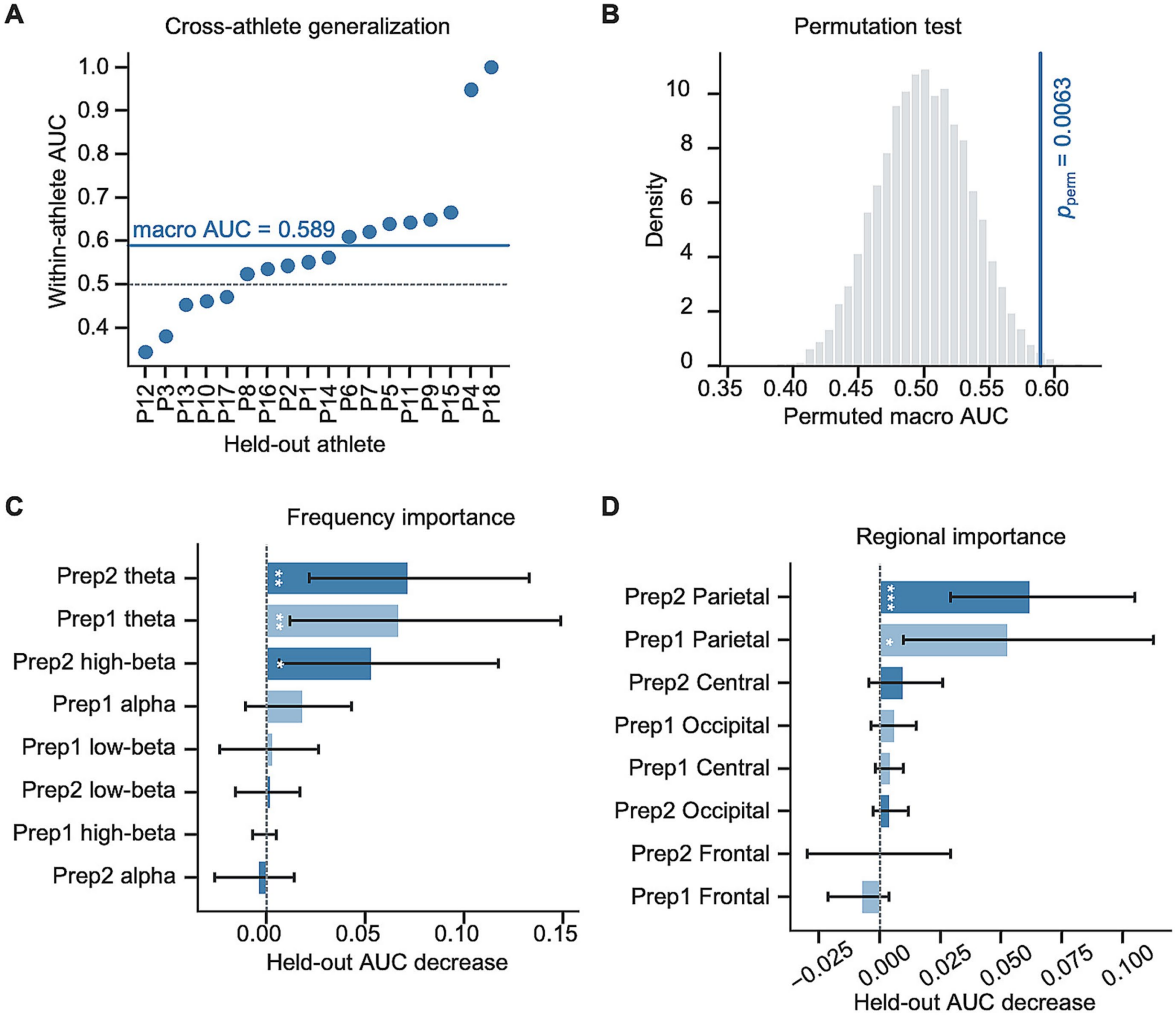
Explainable cross-athlete learning of trial reliability. **(A)** Within-athlete AUC for each held-out athlete. The solid blue line is the macro AUC and the dashed line is chance. **(B)** Null distribution from 10,000 within-athlete label permutations; The blue line is the observed macro AUC. **(C,D)** Held-out grouped permutation importance for stage-frequency and stage-region groups. Positive values indicate loss of predictive information after shuffling; Error bars are athlete-bootstrap from 95% confidence intervals. *p < 0.05, **p < 0.01, and ***p < 0.001.

The primary Prep1-Prep2 multiband model produced macro AUC of 0.589 across 18 evaluable held-out athletes (Figure 3A). Held-out performance varied substantially between athletes, but the observed macro AUC exceeded the within-athlete permutation distribution (*p* = 0.0063; Figure 3B). The result therefore indicates weak but reliable population-shared information that generalized to unseen athletes but not uniformly accurate prediction for every individual.

Held-out grouped permutation importance localized predictive information (Figures 3C,D). Shuffling Prep2 theta features decreased AUC by 0.072 on average (95% athlete-bootstrap CI [0.022, 0.133]; *p* = 0.0039). Prep1 theta produced a similar decrease of 0.067 (95% CI [0.012, 0.149]; *p* = 0.0077), and Prep2 high beta produced a smaller decrease of 0.053 (95% CI [0.006, 0.117]; *p* = 0.0225). Region-wise permutation implicated parietal features in Prep1 (mean decrease = 0.053, 95% CI [0.010, 0.113]; *p* = 0.0112) and, especially, in Prep2 (mean decrease = 0.062, 95% CI [0.029, 0.105]; *p* < 0.001). These uncorrected exploratory importance tests identify information used by the model rather than causal oscillatory or anatomical generators.

Outcome specificity defines the boundary of the decoder. Absolute target-line error did not show a reliable shared mapping. Joint Prep1-Prep2 activity weakly generalized inward-normalized line error (macro Spearman’s *r* = 0.082, 95% CI [0.024, 0.145]; permutation *p* = 0.0127), whereas trial-level compactness remained a trend (macro *r* = 0.043, *p* = 0.059). The strongest evidence therefore concerns whether a serve crossed the target-zone reliability threshold.

### 3.4 Multiscale integration combined complementary predictive information

**Figure 4:**
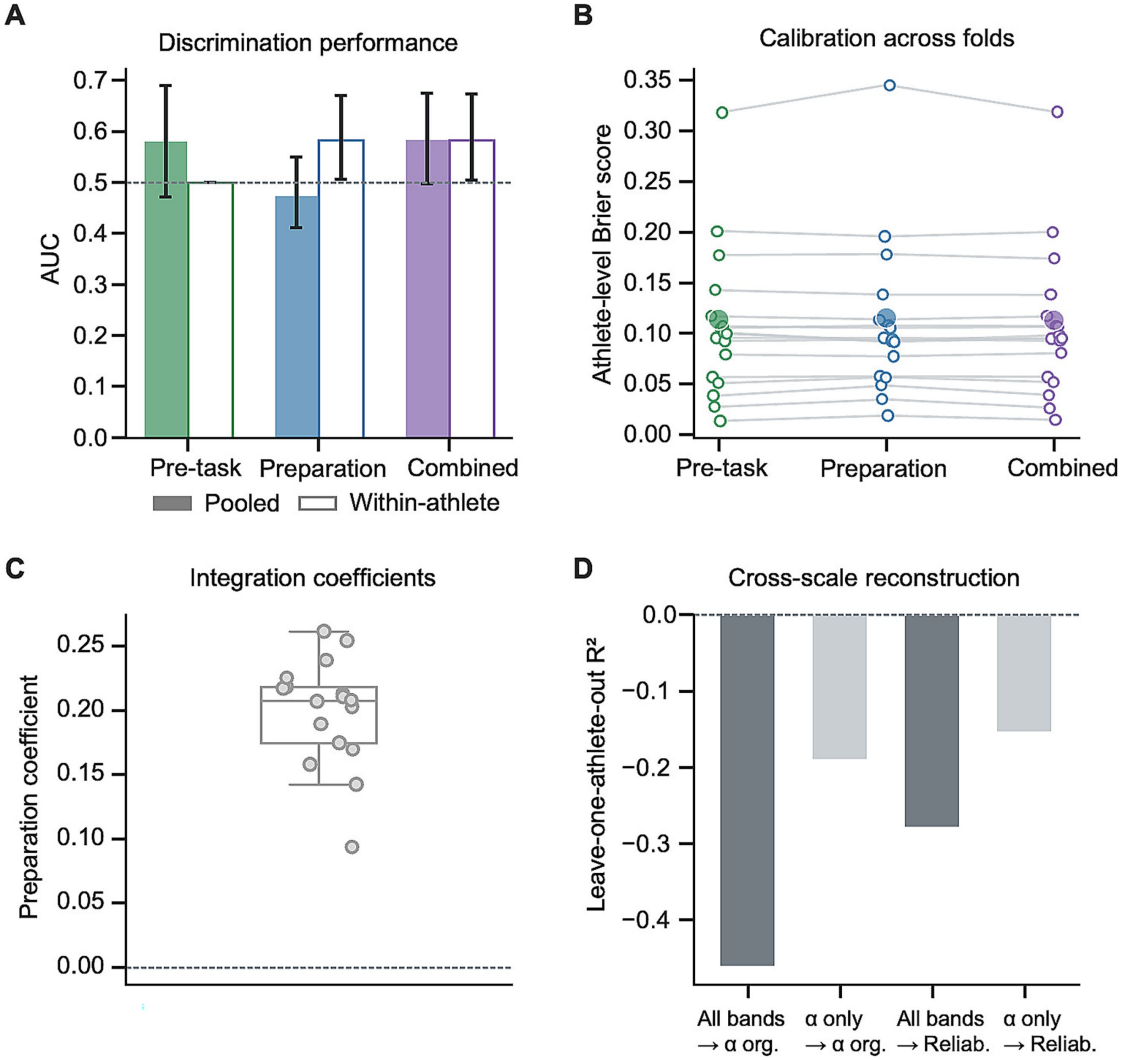
Multiscale brain behavioral integration. **(A)** Pooled and within-athlete AUCs for pre-task, preparation, and combined models; error bars are 95% confidence intervals from athlete-cluster bootstrap samples. **(B)** Fold-level Brier scores; gray lines connect the three models within each held-out athlete. Lower values indicate better calibration. **(C)** Cross-fitted preparation coefficients from the integrated logistic model. **(D)** Leave-one-athlete-out *R*^2^ for reconstruction of pre-task alpha organization and reliability from athlete-average preparation spectra. Negative values indicate performance below an intercept-only held-out prediction.

The pre-task baseline distinguished athletes but remained constant across their trials: it yielded a pooled AUC of 0.583 and a macro within-athlete AUC of 0.500. Preparation EEG ranked trials within athletes (macro AUC = 0.583 in the common 17-athlete subset) but did not discriminate athletes well (pooled AUC = 0.476). The combined model retained within-athlete ranking and restored the pooled AUC to 0.586 (Figure 4A). Corresponding Brier scores were 0.1134 for pre-task only, 0.1148 for preparation only, and 0.1129 for the combined model; fold-level values showed no dramatic calibration gain (Figure 4B). All outer-fold preparation coefficients were positive (Figure 4C), indicating that trial evidence entered the integrated model consistently.

The critical boundary was that athlete-average preparation spectra did not reconstruct the pre-task baseline. Nested leave-one-athlete-out ridge regression produced R2 = −0.46 for all-band summaries and −0.19 for alpha-only regional summaries when predicting alpha organization. The same features also failed to predict overall reliability (R2 = −0.28 and −0.15, respectively; Figure 4D). Thus, pre-task and preparatory EEG were not interchangeable measures along a single athlete-average axis in this dataset. Integration instead combined complementary information operating at different statistical levels.

### 3.5 Sensitivity analyses favored the parsimonious stage model

A five-bin trajectory spanning Prep1, cue, and Prep2 reached macro AUC of 0.558, but its within-athlete permutation test was borderline (*p* = 0.054); cue-only bins were not informative, and the trajectory did not exceed the split-stage model. A two-dimensional probabilistic model did not improve the subject-equal out-of-sample negative log-likelihood, and allowing EEG to predict trial-specific uncertainty worsened performance. These negative and weak findings argue against claiming a complete neural reconstruction of landing distributions and support the narrow reliability model.

## 4 Discussion

The main finding of the present study is that behavioral variability and neural predictive information appear to be organized across the corresponding statistical scales. The largest components of landing-distribution variance reflected reproducible individualized placement patterns, whereas task-relevant performance was more directly captured by reliability and compactness measures. Pre-task alpha organization was associated with between-athlete differences in these outcomes, whereas preparatory EEG carried weak but reliable trial-level information that generalized across athletes. Together, these findings support a multiscale view of skilled action in which different neural signals contribute to different levels of behavioral variability.

The behavioral decomposition was intended to describe how landing distributions varied across athletes before linking those patterns to EEG. In this sense, the PCA results are best read together with the task-defined behavioral descriptors rather than as a strict separation between style and performance. PC1 and PC2 captured the dominant depth and lateral placement tendencies, whereas reliability, compactness, and the smaller PC3-related pattern described the extent to which those landing tendencies satisfied the task constraints. This distinction is important because skilled behavior can preserve stable individual organization while still differing in how consistently the task goal is achieved. In skilled action, variability is reorganized around task constraints rather than eliminated (Krakauer and Mazzoni, 2011; Ranganathan et al., 2020; Wolpert et al., 2011). Target-zone reliability captured whether the primary spatial requirement was met, whereas compactness captured how tightly successful outcomes were stabilized. The PCA and fingerprint analyses therefore highlight an important distinction between dominant behavioral variance and task-relevant variance. Thus, the dominant behavioral dimensions reflected how athletes tended to serve, whereas performance-related dimensions reflected how successfully they satisfied task demands.

The positive association between alpha organization and subsequent performance suggests that athletes differed in their pre-task neural readiness before entering the serving task. Previous studies have linked IAPF and alpha-network dynamics to attentional performance and skilled behavior, although findings across sport domains have not been entirely consistent (Christie et al., 2017; Grandy et al., 2013a; Haegens et al., 2014; Klimesch, 1999, 2012; Zhang et al., 2021). The present results extend this literature by showing that a composite measure incorporating both local oscillatory properties and broader coupling patterns was associated with subsequent reliability and compactness. The index was not intended to isolate a single cognitive state. Rather, it summarized the combination of a relatively faster individual alpha rhythm and lower broad fronto-central alpha coupling before task execution. Sport EEG studies have also reported associations between reduced temporal-frontal alpha coupling and superior self-paced performance, although the functional meaning of this coupling remains debated (Gallicchio et al., 2016; Parr et al., 2021; Raman and Filho, 2024). Because our measure was obtained before task engagement and summarized broad fronto-central coupling rather than task-specific processing, it should not be interpreted as direct evidence of reduced verbal control. Instead, the findings are consistent with the possibility that higher IAPF combined with lower broad alpha coupling reflects a favorable pre-task configuration for subsequent performance. Several theoretical frameworks could account for this association, including neural efficiency, reinvestment, conscious movement processing, and attentional focus perspectives (Beilock and Carr, 2001; Chang and Hung, 2020; Filho et al., 2021; Li and Smith, 2021; Masters and Maxwell, 2008; Masters, 1992; Wulf, 2013). Although the present data do not distinguish among these explanations, they suggest that stable pre-task neural organization may contribute to athlete-level differences in performance.

Preparatory information showed a stage-sensitive functional pattern. Theta activity was informative both before and after directional information became available, whereas an additional high beta contribution emerged only during the post-cue preparation stage. One interpretation is that Prep1 primarily reflected general readiness for action, whereas Prep2 incorporated information related to the upcoming spatial goal while maintaining the task set. The parietal emphasis observed in the model is also compatible with spatial-goal representation and visuomotor integration. This interpretation is broadly consistent with previous reports showing cue-related reorganization of theta, alpha, and beta activity during externally guided sport actions (Christie et al., 2019). The high-beta contribution, however, should be interpreted cautiously. Scalp activity above approximately 20 Hz can overlap with electromyographic activity from the cranial, facial, neck, and postural muscles, particularly during tasks involving movement preparation (Goncharova et al., 2003; Muthukumaraswamy, 2013; Whitham et al., 2007). The exclusion of frontal-polar and temporal channels reduced sensitivity to some artifact sources, but this does not rule out residual myogenic contributions. The Prep2 high-beta effect therefore indicates useful high-frequency information during informed preparation, without resolving whether that information was neural, myogenic, or a mixture of both.

Preparatory EEG carried generalizable trial-level information for athletes excluded from model fitting. Grouped permutation importance was then used to identify which feature groups contributed most consistently to the held-out information. Importantly, the observed effect generalized to athletes excluded from model fitting, indicating that predictive information was not restricted to idiosyncratic individual patterns. This perspective is consistent with predictive-neuroimaging frameworks that emphasize evaluation of unseen individuals and the separation of predictive performance from mechanistic interpretation (Rosenberg et al., 2016; Shen et al., 2017). Although both were behaviorally relevant, athlete-average preparatory spectra failed to reconstruct the pre-task alpha-organization axis. Together with the contrast between pooled and macro AUC, this pattern suggests that pre-task EEG primarily indexed athlete-level performance propensity, whereas preparatory EEG carried trial-specific evidence around that baseline. Rather than reflecting a single cross-timescale neural representation, these signals appeared to provide complementary information on different behavioral scales.

## 5 Conclusion

The present findings suggest that expert action reliability is supported by neural signals operating at different behavioral scales. Pre-task neural organization was associated with between-athlete performance propensity, whereas preparatory activity carried trial-specific evidence that generalized across athletes. Their failure to reconstruct one another, together with their complementary predictive roles, indicates that multiscale integration is more informative than searching for a single universal neural marker. Machine-learning analyses help keep behavioral structure, prediction, and interpretation aligned with the statistical level at which each neural measure was evaluated.

## Data Availability

The de-identified data and analysis code required to reproduce the main findings are available on GitHub: https://github.com/Qiwei-Zhao/pre-task-and-preparatory-EEG-of-expert-action-reliability.
